# CAR T-cell therapy in pediatric acute lymphoblastic leukemia a new paradigm

**DOI:** 10.1097/MS9.0000000000004372

**Published:** 2025-11-15

**Authors:** Asra Amjad, Mir Raza Ali, Muddassir Khalid

**Affiliations:** a Department of Medicine, Islamic International Medical College, Riphah International University, Rawalpindi, Pakistan; b Department of Medicine, Jinnah Postgraduate Medical Centre Karachi, Pakistan; c Department of Medicine, Nishtar Medical University, Multan, Pakistan

*Dear Editor,*

Highlighting both acknowledged developments and novel treatment options is crucial since juvenile acute lymphoblastic leukemia (ALL) is still a hot topic in pediatric oncology. While front-line therapy cures the majority of juvenile ALL cases, relapsed or refractory (R/R) B-cell ALL remains a serious treatment challenge and has a dismal prognosis. Chimeric antigen receptor (CAR) T-cell therapy’s rapid progression from bench to bedside has revolutionized the treatment of R/R juvenile B-ALL^[1,2]^. Building on this historic shift, it is critical to understand how CAR T cells’ molecular mechanism supports their therapeutic efficacy. After a single infusion, CAR T cells – autologous T lymphocytes that have been genetically modified to express a synthetic receptor that recognizes surface antigens on malignant B-cells, most commonly CD19 – produce antigen-directed cytotoxicity and, in many cases, minimal residual diseases-negative remissions^[2,3]^.

Tisagenlecleucel’s major global registration study established proof of concept and supported regulatory approval for pediatric and young adult R/R B-ALL by demonstrating high initial remission rates (about 81% total remission). In a significant portion of patients, longer follow-up reveals persistent remissions^[1,3]^.

However, addressing the practical limits of this therapy is necessary to convert these remarkable early outcomes into long-term success. Despite these achievements, significant restrictions still exist. Standardized grading and prompt multidisciplinary therapy are necessary for acute toxicities, which include immune effector cell-associated neurotoxicity syndrome and cytokine release syndrome, which are particularly severe. Although they have decreased morbidity, consensus grading and changing management algorithms such as IL-6 blockade, corticosteroids, and new agents do not completely eliminate risk^[2,4]^. After CAR T, disease recurrence is still a challenge; known failure reasons include limited CAR T persistence and antigen-negative relapse (CD19 deletion or downregulation). There is ongoing research into methods to counteract antigen escape, including dual-antigen targeting, epitope-spreading techniques, and pharmacologic antigen up-regulation^[5,6]^.

Beyond the therapeutic challenges, the more general access issue highlights whether CAR T therapy can be implemented globally in an equitable manner. The worldwide impact of CAR T treatment is also limited by pricing and accessibility. In many locations, particularly in), supply is restricted by manufacturing complexity, long vein-to-vein intervals, and high per-patient costs. Reviews of “off-the-shelf” allogeneic CAR-T techniques and real-world experiences from LMIC centers show possible avenues to increase access, but they also show that infrastructure, legal, and technical barriers still exist^[7,8]^.

Despite this, the sector is developing quickly thanks to inventions that get beyond logistical and biological obstacles. Promising breakthroughs for the future include allogeneic “off-the-shelf” products to cut costs and expedite time to therapy, dual-targeting CAR constructs (e.g., CD19/CD22) to prevent antigen-escape, and tailored constructs and manufacturing advancements to increase persistence and reduce toxicity. To turn CAR T’s promise into long-lasting treatments for more children globally, head-to-head and combination studies, coordinated toxicity mitigation techniques, ongoing long-term follow-up, and global equity initiatives will be crucial^[3,5–8]^.

In the end, advancements in CAR T therapy demonstrate both scientific inventiveness and a duty to guarantee that these discoveries benefit kids from all socioeconomic backgrounds. In conclusion, the CAR T-cell therapy has already revolutionized R/R pediatric B-ALL care; but, in order to establish it as the gold standard of care for kids worldwide, we need to combine scientific advancement with practical answers to the problems of toxicity, durability, cost, and accessibility.

TITAN Guidelines: This manuscript is in compliant to TITAN Guidelines, 2025, declaring no use of AI^[9]^.

## Data Availability

Not applicable.
